# Diagnostic Clues to Platypnoea Orthodexia Syndrome

**DOI:** 10.7759/cureus.104221

**Published:** 2026-02-25

**Authors:** Utsah Bhattacharya, Silvia De Andres Quevedo, Zoha Iftikhar, Bassam Fallouh, Eslam Abdelaziz

**Affiliations:** 1 General Surgery, William Harvey Hospital, East Kent Hospitals University National Health Service (NHS) Foundation Trust (EKHUFT), Ashford, GBR; 2 Acute Medicine, Queen Elizabeth The Queen Mother Hospital, East Kent Hospitals University National Health Service (NHS) Foundation Trust (EKHUFT), Margate, GBR; 3 Medical Education, William Harvey Hospital, East Kent Hospitals University National Health Service (NHS) Foundation Trust (EKHUFT), Ashford, GBR; 4 Acute Medicine, William Harvey Hospital, East Kent Hospitals University National Health Service (NHS) Foundation Trust (EKHUFT), Ashford, GBR

**Keywords:** hypoxia, patent foramen ovale (pfo), pfo closure device, platypnoea orthodexia syndrome, pos

## Abstract

Platypnoea orthodeoxia syndrome is a rare cause of positional dyspnoea, characterised by breathlessness and arterial desaturation in the upright position that improves with lying flat. This phenomenon is typically due to a right to left shunt, most commonly cardiac, that allows deoxygenated blood to bypass the pulmonary circulation, therefore reducing oxygen saturation in the systemic circulation. This shunt is accentuated by postural changes, worsening hypoxaemia when the patient sits or stands, and ameliorated by recumbency, which alters intracardiac pressures and improves oxygenation. Despite its clinical significance, platypnoea orthodeoxia syndrome remains underdiagnosed.

We are presenting two patients with platypnoea orthodeoxia syndrome. Both patients were found to have significant positional hypoxaemia in the upright position that was found to be improving on recumbency, both of whom were subsequently diagnosed with a patent foramen ovale with right to left flow, in addition to aortic dilatation as the underlying cause. They both underwent a series of investigations to confirm orthodeoxia, which is the term referring to dropping of oxygen saturation in an upright position, as well as to rule out other causes of desaturation. They both went on to have minimally invasive closure of the patent foramen ovale later in the course of their management with the resolution of symptoms and improvement in oxygen saturation.

## Introduction

Platypnoea orthodeoxia syndrome (POS) is a rare clinical condition characterised by dyspnoea and arterial desaturation that worsens in the upright position and improves on assuming a supine posture. Although described nearly a century ago, the underlying pathophysiology of POS remains complex and multifactorial.

The mechanisms of POS can be broadly categorised into intracardiac shunts, extracardiac (pulmonary) shunts, combined cardiopulmonary causes, and miscellaneous aetiologies. In a review of 239 reported cases, intracardiac shunts accounted for 87% of cases [1]. The most implicated abnormalities include patent foramen ovale (PFO), atrial septal defect (ASD), and atrial septal aneurysm (ASA), which permit interatrial shunting. In most cases, an additional anatomical or functional factor is required to facilitate right-to-left shunting in the upright position. These secondary factors may cause deformation or increased mobility of the atrial septum, or elevated right atrial pressure, thereby redirecting venous blood into the systemic circulation. Reported contributors include aortic dilatation - either aneurysmal or distorted - present in approximately 20% of cases [2], as well as pneumonectomy, diaphragmatic paralysis, and a prominent Eustachian valve.

Autonomic influences may also play a role. Acute sympathetic activation has been shown to increase right-to-left shunting in patients with PFO-associated stroke through elevations in systemic blood pressure and intracardiac pressures, or via increased intrathoracic pressure resulting in a Valsalva-like effect [3]. It is plausible that similar mechanisms may precipitate or exacerbate POS in the presence of an interatrial communication.

POS was first described in 1949 in a patient with a post-traumatic intrathoracic arteriovenous shunt. Despite this early recognition, the condition remains underreported; a 2017 review identified only 239 cases across 150 publications, excluding patients with complex congenital heart disease [1]. More recently, a retrospective study published in 2025 reported a median pre-procedural hospital stay of 40 days among 11 patients with POS and PFO [4]. These findings highlight the diagnostic challenges associated with POS and suggest that the condition is likely underdiagnosed due to limited clinical awareness and the complexity of its evaluation.

This case report aims to improve the clinical recognition of POS and to underline the importance of considering this diagnosis in patients with otherwise unexplained positional hypoxaemia.

## Case presentation

Case 1

A 58-year-old female patient with a past medical history of hypertension and hypothyroidism attended the Emergency Department (ED) with a sudden-onset, severe headache. Examination revealed no neurological deficit and was otherwise unremarkable, with equal bilateral air entry in both lungs with no added sounds and no murmurs on cardiac auscultation. However, on the first day of admission, the patient started feeling breathless, and her oxygen saturation dropped as low as 70%. Following this, she was admitted to the intensive treatment unit (ITU), where she required high-flow nasal oxygen (HFNO) and was briefly on continuous positive airway pressure (CPAP) to maintain target oxygen saturations. She was subsequently weaned off supplemental oxygen before being discharged to the ward. She was reviewed by multiple teams, including the critical care outreach team (CCOT), intensive care unit (ICU), and cardiology, respiratory, and neurology teams.

**Figure 1:**
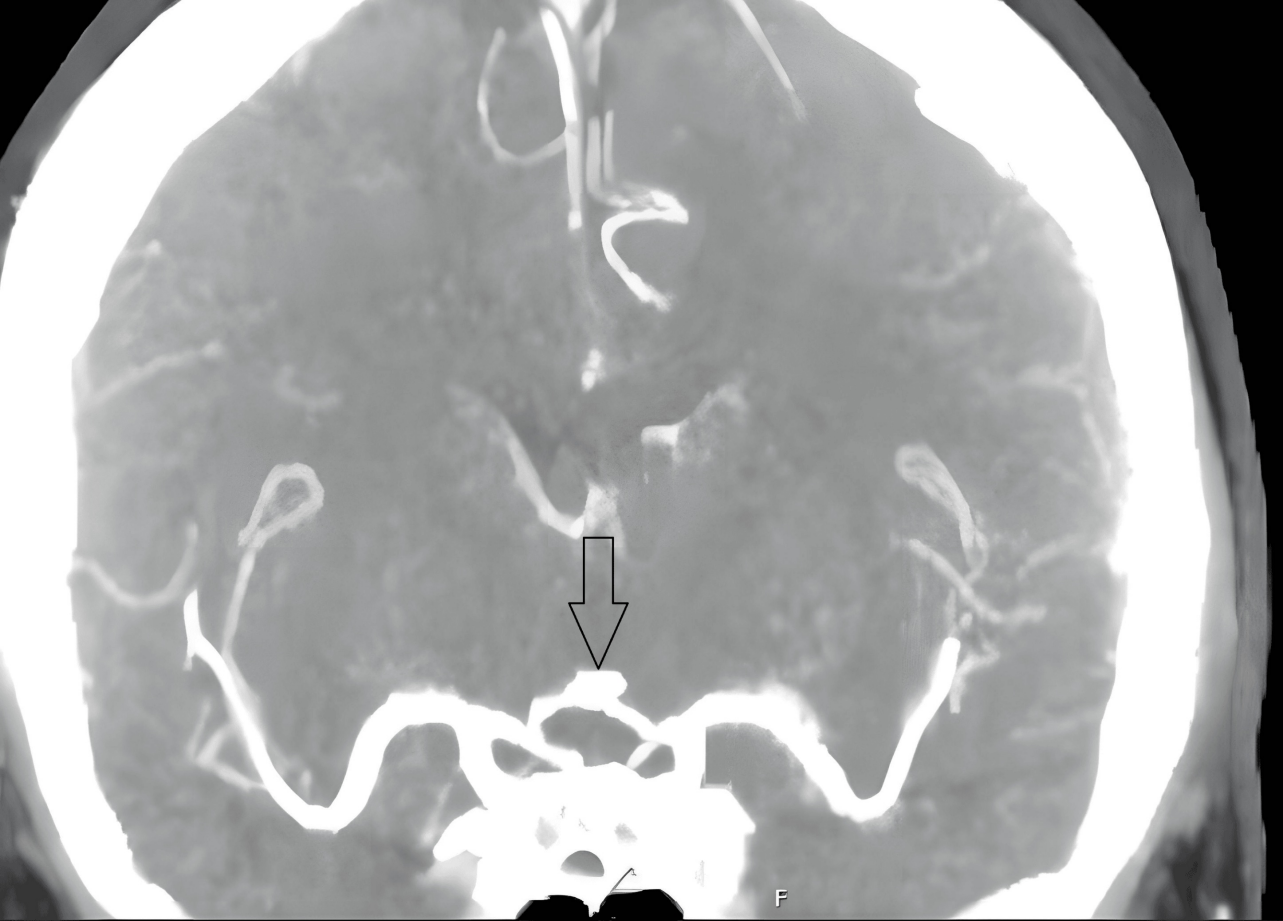
CTA Circle of Willis: 4 mm basilar tip aneurysm. Possible minor focal dilatation also seen in the left A2 segment measuring 1.7 mm (arrow). The intracranial segments of the internal carotid arteries and the major vessels forming the Circle of Willis and their branches are of normal calibre and are patent.

As part of investigations for the headache, she underwent a computed tomography (CT) head and a CT intracranial angiogram (Figure 1), which showed no intracranial bleed but identified a 4 mm basilar tip aneurysm. She also had a lumbar puncture that was positive for xanthochromia, leading to a diagnosis of subarachnoid haemorrhage (SAH).

Serial arterial blood gases (ABGs) (as depicted below in Table 1) on room air were taken while the patient was sitting and lying down, confirming positional hypoxaemia and type 1 respiratory failure in the upright position with a drop in partial pressure of oxygen (PaO₂) from 11.7 to 7.3 kilopascals (kPa) and oxygen saturation (SpO₂) from 96.1% to 88.2%, respectively, within minutes between samples taken from the same arterial line.

**Table 1:** Serial arterial blood gases (ABGs) on room air were taken while sitting and lying down Serial arterial blood gases (ABGs) on room air were taken while sitting and lying down, confirming positional hypoxaemia and type 1 respiratory failure in the upright position with a drop in partial pressure of oxygen (PaO₂) and oxygen saturation (SpO₂) from 11.7 to 7.3 kilopascals (kPa) and from 96.1% to 88.2%, respectively, within minutes between samples taken from the same arterial line. FiO_2_: Fraction of inspired oxygen.

Parameters	On admission	Day 2	Day 2	Day 3	Day 4	Day 7	Day 10
pH	7.46	7.38	7.42	7.38	7.42	7.39	7.41
pO_2_	7.3	9.8	15.2	9.2	13.9	11.1	10.4
pCO_2_	3.6	4.6	4.0	4.5	4.6	4.6	5.1
Saturation	85.5	96.2	99.9	95.6	99.7	98	98
FiO_2_	Not recorded	60	50	30	24	21	21

**Figure 2:**
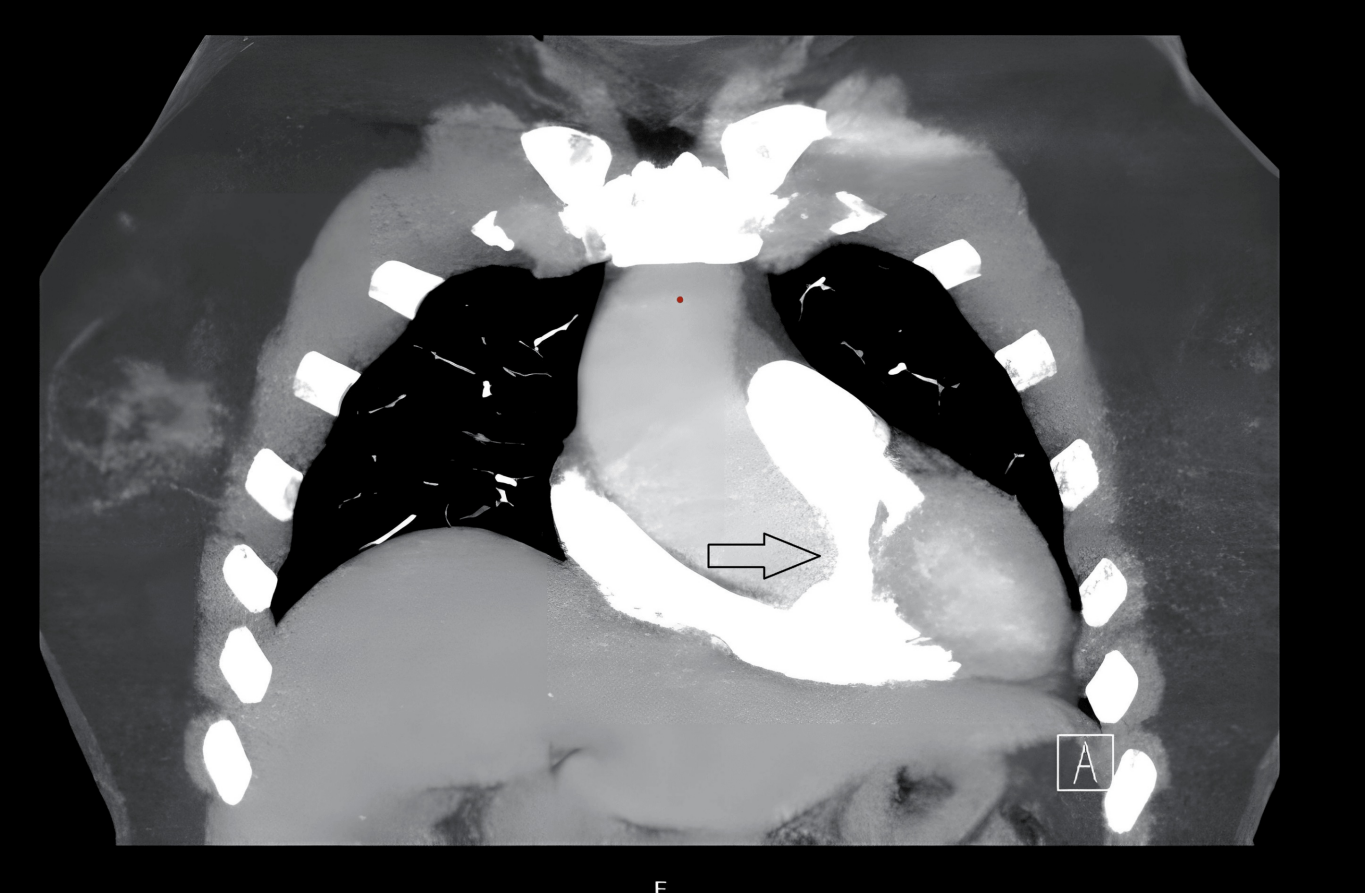
CT angiogram aorta and the arrow depicts aneurysmal dilatation of the ascending aorta that measures 4 cm.

To investigate the hypoxaemia, she underwent a CT pulmonary and aortic angiogram (Figure 2), which revealed a 45 mm ascending aortic dilatation. A ventilation-perfusion (V/Q) scan was also normal.

**Figure 3:**
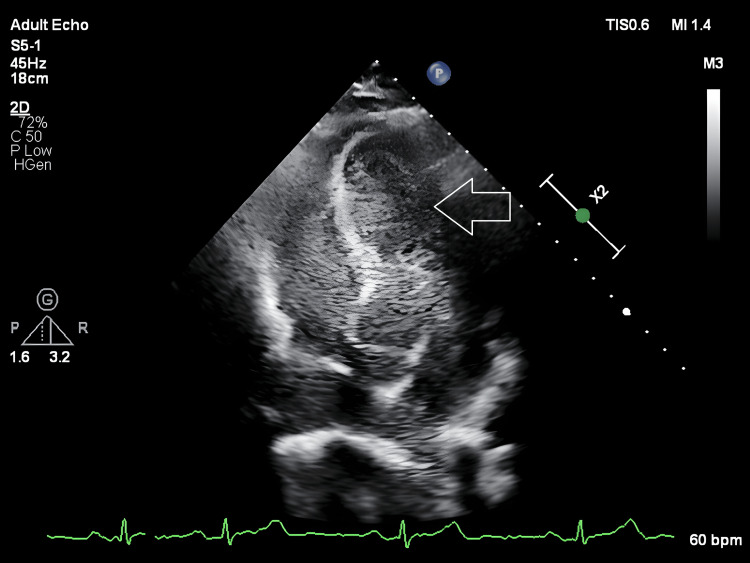
A still image from bubble echocardiography showing opacification of all four chambers (arrow).

Contrast (bubble) echocardiography (Figure 3) was performed at rest, with cough/sniff manoeuvres, and during Valsalva manoeuvre. No shunt was observed at rest or with cough/sniff. During Valsalva manoeuvre, there was immediate and complete opacification of the left ventricle, consistent with a Grade 4 right-to-left shunt.

The patient was discharged home but later complained of ongoing breathlessness. Pulmonary function tests were performed and were normal. She was therefore brought back for a transoesophageal echocardiogram (TOE) followed by an elective minimally invasive PFO closure [5] and is currently being followed up by the adult congenital heart disease (ACHD) team. Her symptoms improved markedly following the procedure.

Case 2

A 63-year-old man with hypertension, a significant smoking history, and a previous knee replacement presented to his pre-assessment appointment for cataract surgery. He complained of shortness of breath and was found to have severe hypoxaemia with peripheral oxygen saturation (SpO₂) of 69%. He was transferred to the Emergency Department (ED), where he was admitted to the resuscitation area. Clinical examination was unremarkable, showing equal bilateral air entry with no added sounds on chest auscultation and normal heart sounds without murmurs.

It is worth noting that his symptoms were out of proportion to the degree of hypoxaemia, which improved only marginally to 78% on 15 litres per minute of oxygen via a non-rebreather mask. The patient was subsequently admitted to the ICU, where he was started on high-flow nasal oxygen (HFNO) at 60 litres per minute with a fraction of inspired oxygen (FiO₂) of 93% to achieve an oxygen saturation (SpO₂) of 85% when sitting up. This improved to 99% when lying flat.

To further investigate the severe hypoxaemia, a series of investigations were undertaken with a multidisciplinary team approach involving cardiologists, pulmonologists, and intensivists. Serial ABGs performed in sitting and supine positions demonstrated improvement in all parameters on lying flat, with SpO₂, PaO₂, and oxyhaemoglobin (O₂Hb) improving from 89.3% to 99%, 7.2 to 23.8 kilopascals (kPa), and 87.6% to 97.2%, respectively.

A CT pulmonary angiogram (CTPA) and a high-resolution CT of the chest showed no evidence of pulmonary embolism (PE) or arteriovenous malformations (AVMs) but revealed emphysematous changes in both lungs. A CT angiogram of the aorta and both carotid arteries showed dilatation of the descending thoracic aortic arch measuring 4 cm. An abdominal ultrasound was performed to rule out hepatopulmonary syndrome (HPS) as a cause for POS and was normal.

Finally, a bubble echocardiogram identified a patent foramen ovale (PFO) with a grade 4 right-to-left shunt and complete opacification of both right and left heart chambers, along with a hyperkinetic interatrial septum.

**Figure 4:**
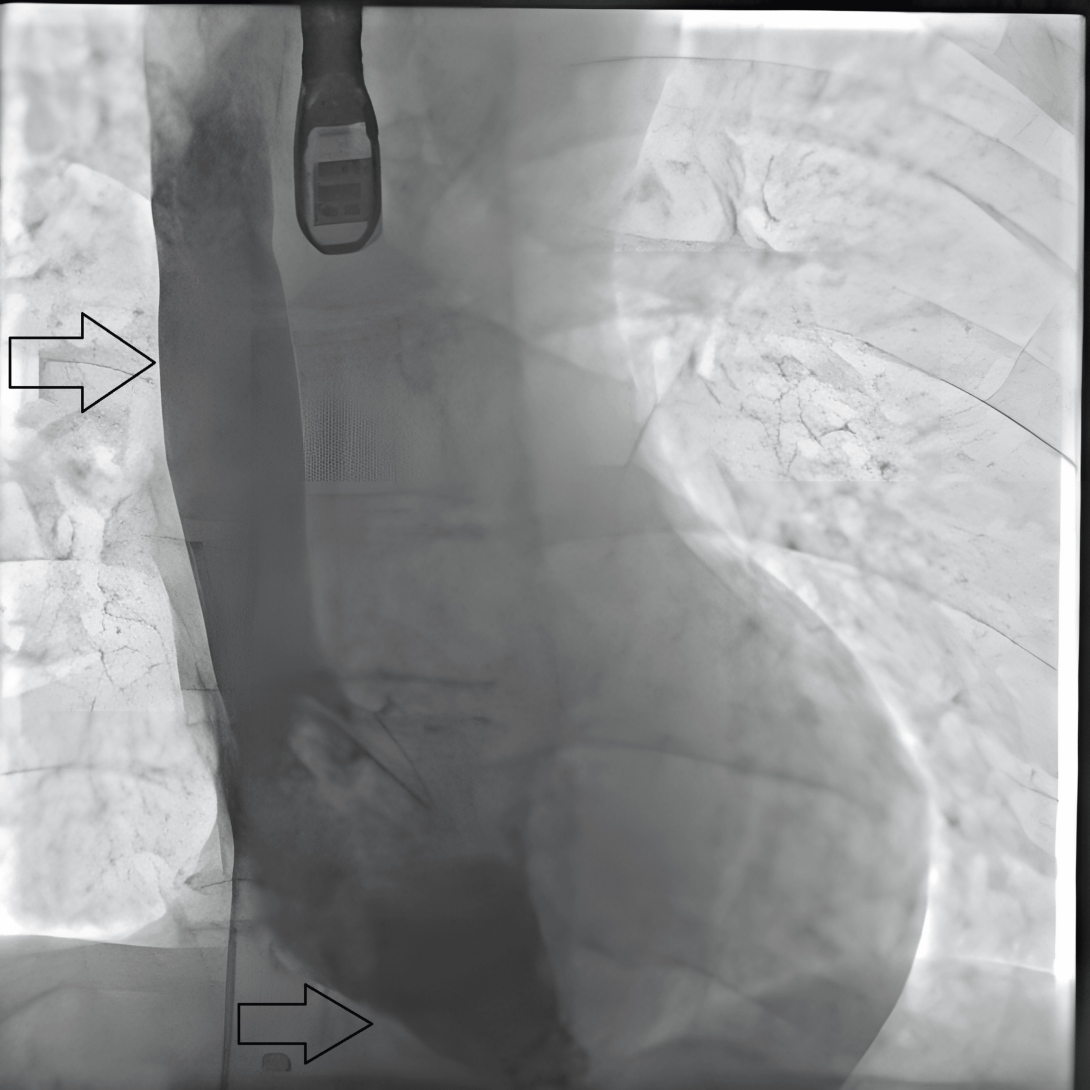
Post-procedural result: Still image from transcatheter procedure with the arrow showing contrast flowing from superior vena cava to right ventricle with no right to left shunt.

**Figure 5:**
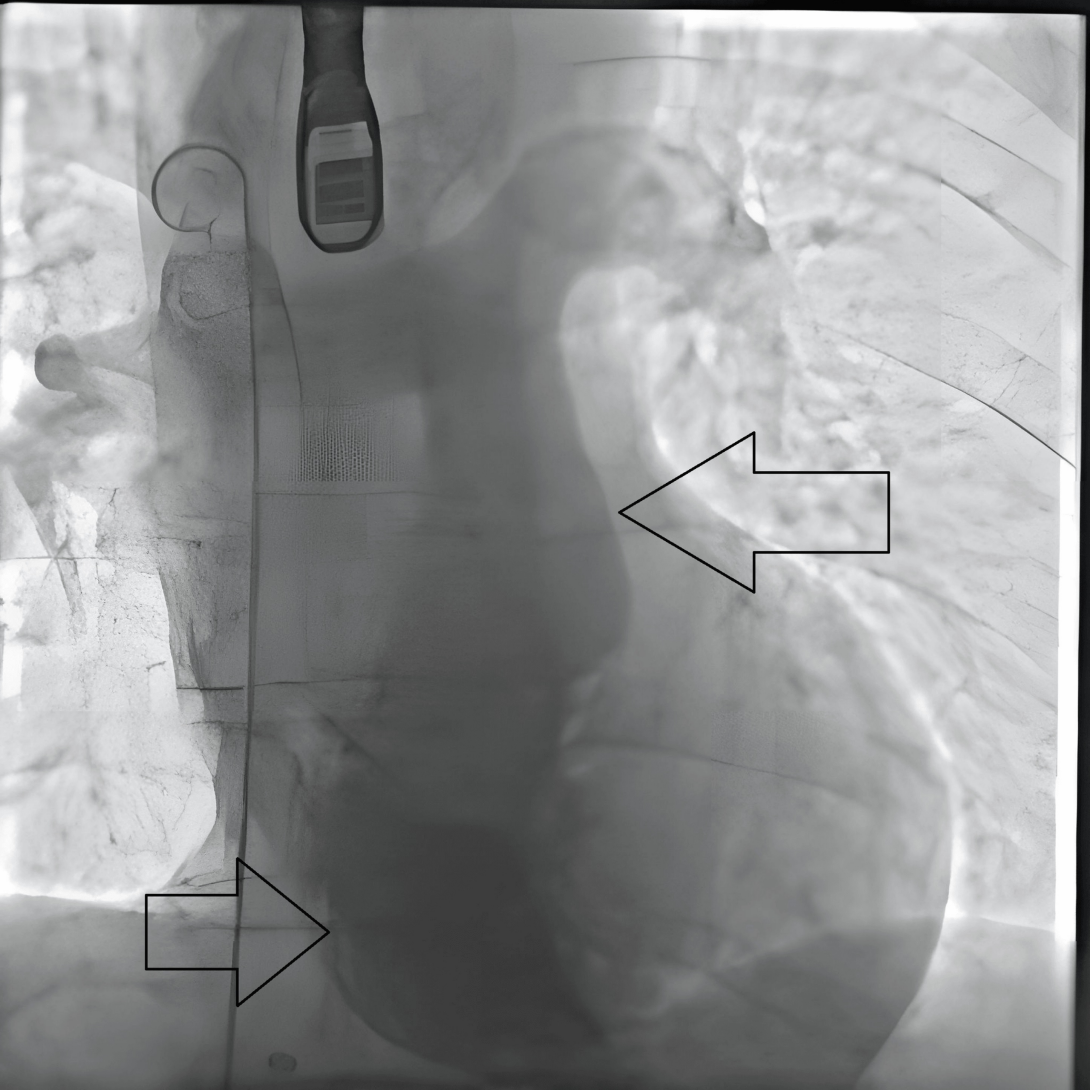
Post-procedural result: Still image from transcatheter procedure with the arrow showing contrast flowing from right ventricle to pulmonary trunk

Due to the patient’s ongoing high oxygen requirements, he was transferred to a tertiary centre with an adult congenital heart disease (ACHD) team, where he underwent a transcatheter PFO closure (Figures 4, 5). Post-procedure bubble echocardiography demonstrated no evidence of a residual right-to-left shunt. Importantly, his oxygen saturations improved to 92% on room air, and he reported symptomatic relief and satisfaction with the resolution of his previously unrecognised condition.

## Discussion

POS is an uncommon and frequently underrecognised clinical condition characterised by positional dyspnoea and hypoxaemia that worsens in the upright position and improves when lying supine. It can arise from multiple etiologies, including intracardiac shunts such as PFO, pulmonary arteriovenous malformations, and ventilation-perfusion mismatch. The diagnostic challenge of POS, particularly when symptoms are disproportionate to routine examinations, highlights the need for multidisciplinary evaluation involving cardiology, respiratory, and critical care teams.

The pathophysiology of POS with intracardiac shunts like PFO involves abnormal right-to-left shunting of deoxygenated blood without elevated right heart pressures. Secondary factors such as aortic dilatation or distortion can alter right atrial anatomy, promoting shunting when upright. One reported case combined a basilar tip aneurysm and aortic dilatation, while another case showed emphysematous lung disease worsening ventilation-perfusion mismatch, contributing further to hypoxaemia [1].

Both patients demonstrated high-grade right-to-left shunting through a patent foramen ovale in the presence of aortic dilatation, a recognised anatomical contributor that can facilitate interatrial flow in POS. In Case 1, subarachnoid haemorrhage may have acted as a physiological trigger. The associated catecholamine surge is known to produce acute haemodynamic alterations, including changes in myocardial function and systemic pressures, which may transiently modify intracardiac and intrathoracic pressure relationships and unmask a previously asymptomatic shunt [6].

In Case 2, emphysematous lung disease likely contributed through worsening ventilation perfusion mismatch in the upright position. Gravity-dependent redistribution of pulmonary blood flow, combined with reduced pulmonary arterial flow to upper lung zones, may create functional dead space physiology and increase physiological right-to-left shunting, thereby exacerbating positional hypoxaemia, as described in prior reviews of POS [1].

Both patients underwent transcatheter PFO closure with subsequent improvement in positional symptoms and oxygenation.

Bubble echocardiography with provocative maneuvers (e.g., Valsalva) is essential for detecting and grading shunts, supporting POS diagnosis. Serial arterial blood gases comparing sitting and supine positions quantify positional hypoxaemia, and imaging like CT pulmonary angiogram, ventilation-perfusion scans, and pulmonary function tests exclude other causes such as pulmonary embolism or hepatopulmonary syndrome.

Transcatheter PFO closure effectively resolves hypoxaemia and symptoms in most cases, improving oxygen saturations and quality of life. Some patients may persist with symptoms post-closure, requiring evaluation for multifactorial causes including chronic lung disease or other comorbidities.

Another retrospective study in 2025 showed that the median length of pre-procedure hospital stays for patients with POS and PFO, in a cohort of 11 patients, was 40 days [4]. The study highlighted that the median length of stay before the procedure was 40 days, reflecting the diagnostic delay typical of this syndrome. Potentially being diagnosed due to lack of knowledge of the condition or the challenging process of the diagnosis.

Clinicians should maintain a high index of suspicion for POS in patients with unexplained hypoxaemia and positional symptoms. Early recognition and prompt management, including closure of intracardiac shunts where appropriate, are vital to improving patient outcomes. Larger studies are needed to assess long-term outcomes and adjunctive therapies for multifactorial cases [1].

## Conclusions

Early recognition of POS is crucial due to its subtle and positional symptoms. Clinicians should assess oxygenation both upright and supine using pulse oximetry and arterial blood gases, as positional hypoxaemia is a hallmark feature. Bubble echocardiography is essential to detect and grade intracardiac shunts, while additional imaging such as chest CT and ventilation-perfusion scans can identify extracardiac causes. Definitive management depends on the underlying etiology, with percutaneous PFO closure effectively improving symptoms and oxygenation. Patients require follow-up to monitor for residual or recurrent hypoxaemia and to address any contributing comorbidities.
